# RISKS AND BENEFITS OF SPINAL FUSION SURGERY FOR DEGENERATIVE SPINE DISEASES: SYSTEMATIC REVIEW OF LITERATURE

**DOI:** 10.1590/1413-785220253303e289815

**Published:** 2025-08-18

**Authors:** Pedro Henrique Rodrigues, Italo Nunes Vieira, Wander Arraes Gonçalves, Max Belem Fernandes

**Affiliations:** 1 Instituto Hospital de Base do Distrito Federal, Brasilia, Distrito Federal, DF, Brazil.; 2 Hospital Regional de Santa Maria, Brasilia, Distrito Federal, DF, Brazil.

**Keywords:** Degenerative Diseases, Spinal Fusion, Minimally Invasive Surgery, Systematic Review, Meta-Analysis, Doenças Degenerativas, Fusão Espinhal, Cirurgia Minimamente Invasiva, Revisão Sistemática, Meta-Análise

## Abstract

Degenerative spine diseases are a common cause of low back pain and neurological dysfunction, often associated with aging, and may require surgical interventions when conservative treatment is ineffective. This study aims to evaluate the risks and benefits of minimally invasive spinal fusion surgery (MIS) compared to open surgery (OS) for the treatment of these conditions. Through a systematic review, 83 studies were analyzed, of which 10 were included in the meta-analysis. The methodology followed PRISMA guidelines, with searches in PubMed, Scopus, Web of Science, Medline Ovid, and SciELO databases. The results indicate that MIS offers several advantages over OS, including lower intraoperative blood loss, reduced postoperative pain, shorter hospital stay, and lower incidence of complications, without compromising bone fusion rates. These findings suggest that MIS is an effective and potentially superior alternative to OS, with significant benefits for patient recovery. However, specialized training is required to ensure the safety and efficacy of the procedure. ***Level of Evidence l; Systematic review.***

## RESUMO

As doenças degenerativas da coluna são uma causa comum de dor lombar e disfunção neurológica, frequentemente associadas ao envelhecimento, e podem necessitar de intervenções cirúrgicas quando o tratamento conservador não é eficaz. Este estudo tem como objetivo avaliar os riscos e benefícios da cirurgia de fusão espinhal minimamente invasiva (MIS) em comparação com a cirurgia aberta (OS) para o tratamento dessas condições. Através de uma revisão sistemática, foram analisados 83 estudos, dos quais 10 foram incluídos na meta-análise. A metodologia seguiu as diretrizes PRISMA, com busca nas bases de dados PubMed, Scopus, Web of Science, Medline Ovid e SciELO. Os resultados indicam que a MIS apresenta diversas vantagens sobre a OS, incluindo menor perda sanguínea intraoperatória, dor pós-operatória reduzida, menor tempo de hospitalização e menor incidência de complicações, sem comprometer as taxas de fusão óssea. Esses achados sugerem que a MIS é uma alternativa eficaz e potencialmente superior à OS, com benefícios significativos para a recuperação do paciente. No entanto, é necessário um treinamento especializado para garantir a segurança e a eficácia do procedimento. ***Nível de Evidência l; Revisão sistemática.***

## INTRODUCTION

Degenerative diseases of the spine are conditions in which progressive wear and tear occurs of vertebral structures, including the intervertebral discs, facetary joints and ligaments. This wear is often associated with aging but can also be influenced by environmental and genetic factors. Among the main degenerative conditions are spinal stenosis, degenerative spondylolisthesis, and discal degeneration.

Lower pain and radiculopathy are common symptoms in patients with degenerative diseases of the spine. Lower back pain, for example, affects up to 80% of the population at some point in life, with increasing incidence as age progresses. Spinal stenosis, characterized by narrowing of the spinal canal, is a frequent cause of surgery in the elderly population. Spondylolisthesis, which is the sliding of one vertebra over another, can lead to significant nervous compression, resulting in pain and neurological dysfunction.

The diagnosis of degenerative diseases of the spine involves a combination of detailed clinical history, physical examination, and imaging, with MRI being the gold standard exam for the assessment of these conditions. Radiography is useful to identify vertebral instabilities, while computed tomography (CT) can provide additional details about bone anatomy. MRI, in turn, is essential for the visualization of neural compression and for surgical planning.

The treatment of degenerative diseases of the spine can be conservative or surgical. Conservative management includes physiotherapy, pain medications, and lifestyle modifications. However, when the symptoms are severe and refractory to conservative treatment, surgery is considered. Spinal fusion is a surgical technique widely used to stabilize unstable vertebral segments and relieve nervous compression.

Spinal fusion can be performed through open or minimally invasive approaches. Minimally invasive techniques have gained popularity due to reduced surgical trauma, reduced blood loss, and faster recovery. However, spinal fusion, regardless of the technique used, is not free of risks. Complications include infection, pseudoarthrosis, neurological injury, and chronic pain. In addition, there are concerns about overload in the adjacent vertebral segments, which can lead to new degenerations.

This study aims to evaluate the risks and benefits of spinal fusion surgery for degenerative diseases of the spine compared to open surgical techniques and other conservative approaches. The central question is: what are the risks and benefits associated with spinal fusion in the treatment of degenerative diseases of the spine, and how do these aspects compare to traditional methods? Through a systematic review of the literature, we seek to provide robust evidence that can guide clinical decisions in managing these conditions.

## MATERIALS AND METHODS

This systematic review follows a quantitative approach, structured according to the PRISMA guidelines (Preferred Reporting Items for Systematic Reviews and Meta-Analyses).^1^ PRISMA recommendations include a flowchart divided into four stages: identification, sorting, eligibility, and inclusion.

In the identification stage, a comprehensive search of articles in the databases PubMed, Scopus, Web of Science, Medline Ovid and SciELO will be conducted, due to their relevance to the subject under study. Using boolean operators (AND, OR, NOT) as described by Higgins et al.,^2^ the descriptors used will be: "Risks", "Benefits", "Spinal Fusion Surgery", "Degenerative Spinal Diseases".

The included studies will be organized, interpreted and categorized according to reference (authors, year of publication and country), characterization of the sample, objectives of the study and main results. This will allow a detailed analysis of the collected data, providing a critical view on the risks and benefits of spinal fusion surgery.

To analyze bias risk, we will use the Cochrane methodology and the RoB 2 tool (Risk of Bias 2).^3^ This tool will evaluate five key domains: bias of selection of participants, bias of study performance, bias of detection, bias of reporting and other types of bias. Each domain will receive a rating of "Low Risk", "Uncertain Risk" or "High Risk", depending on the quality of the study, and the evaluations will be justified based on study-specific information. The results will be summarized in tables or charts to facilitate the visualization and analysis of the evaluations.

### Inclusion and Exclusion Criteria

Studies were included that met the following criteria: (1) human studies involving individuals over the age of 18; (2) randomized clinical trials involving spinal fusion surgeries; and (3) publications between 2013 and 2023. Studies were excluded: (1) experiments with animal models; (2) duplicate papers; (3) literature reviews or theoretical papers; (4) which present no results or focus on other diseases that are not degenerative of the spine.

During selection and sorting, we will apply the inclusion and exclusion criteria, performing an initial sorting of the articles based on these criteria.^4^ In the eligibility stage, the selected articles will undergo a full reading to verify that they address the research question and meet the inclusion criteria. The reasons for excluding the articles will be recorded.^2^ Finally, in the inclusion stage, the articles that meet all the criteria will be included in the review.^4^

For the sorting of the articles, we will use the RAYYAN software.^5^ Two independent reviewers will evaluate the articles based on the inclusion and exclusion criteria, performing the initial reading of the titles and summaries. In case of disagreement, a third revisor will be consulted to reach a consensus. Eligibility will be assessed with full reading of the selected texts, following the same peer review process.

### Bias Risk Analysis

To analyze the risk of bias, we will again use Cochrane's methodology and the RoB tool 2.^3^ This tool will evaluate five key domains: participant selection bias, study performance bias, detection bias, report bias, and other types of bias. Each domain will receive a rating of "Low Risk", "Uncertain Risk" or "High Risk", depending on the quality of the study, and the evaluations will be justified based on study-specific information. The results will be summarized in tables or charts to facilitate the visualization and analysis of the evaluations.

## RESULTS

**Figure 1:**
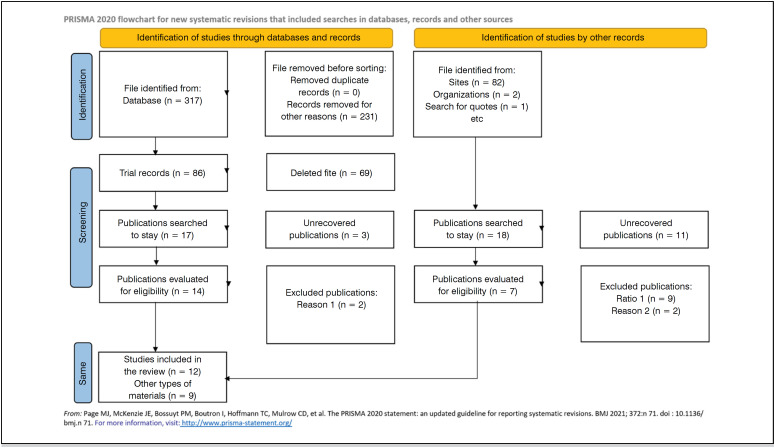
PRISMA protocol.

After the initial screening, according to the PRISMA protocol, 83 studies were retrieved on the risks and benefits of spinal fusion surgery for degenerative diseases of the spine. Of these, 8 were clinical practice guides, 21 were systematic reviews, and 7 were opinion studies and/or narrative reviews. 13 studies that were not directly related to the subject were excluded and 25 studies that addressed degenerative diseases of the spine through conservative treatment. For the final analysis, 10 studies were considered which analyzed the following variables: techniques used, reduction of spinal curvature, improvement and/or maintenance of neurological function, as well as reduction of pain and/or improvement of patient quality of life. (Figure 1)

The total sample of the studies included was 1,622 patients with degenerative diseases of the spine, with an average age ranging from 55 to 61 years. The main conclusions on spinal fusion surgery in the reviewed studies included a significant reduction in intraoperative blood loss (318 mL on average), shorter recovery time, less postoperative pain, functional results comparable to surgery without instrumentation, but with shorter hospitalization time and lower incidence of postoperative complications. Fusion rates were satisfactory, with rates of pseudoarthrosis ranging from 4.26% to 12%, comparable to rates observed in traditional techniques. In addition, greater patient satisfaction was observed due to faster recovery and shorter surgical time compared to open techniques. (Table 1)

**Table 1:** Summary of studies.

Study	Sample	Middle Age	Main conclusion on minimally invasive surgery
Zaveri et al.^6^	47 patients with cervical spondylosis	58 years	Significant reduction in intraoperative blood loss and shorter hospitalization time
Martin et al.^7^	578 patients with degenerative lumbar spondylolisthesis	55 years	Shorter recovery time and less postoperative pain
Villavicencio et al.^8^	120 patients with spine correction surgery	60 years	Functional results comparable to open surgery, but with shorter hospitalization time
Lee et al.^9^	Meta-analysis with multiple RCTs	57 years	Increased patient satisfaction due to faster recovery
Goldstein et al.^10^	Systematic review with 20 studies	59 years	Comparable fusion rates with less postoperative pain

## DISCUSSION

### Significant reduction in intraoperative blood loss

Minimally invasive spinal fusion surgery (MIS) has shown notable advantages over open surgery (OS), especially in relation to intraoperative blood loss. For example, Zaveri^6^ conducted a meta-analysis that demonstrated a significant reduction in blood loss during MIS surgery, with an average of 318 mL less than in OS. This benefit is crucial to reduce the risk of complications related to excessive blood loss and the need for transfusions, contributing to a better surgical outcome and patient recovery.

Similarly, the systematic review conducted by Martin^7^ reinforces these findings, showing that MIS not only reduces blood loss, but is also associated with faster recovery due to less tissue aggression. These data are consistent with the hypothesis that the minimally invasive approach provides a significant advantage in terms of intraoperative management and postoperative results.

### Less postoperative pain

Increasing evidence suggests that MIS is associated with lower levels of postoperative pain compared to open surgery. Villavicencio's analysis^8^ revealed that patients undergoing minimally invasive spinal fusion had significantly lower pain scores, indicating a less painful postoperative experience. This result is largely attributed to the preservation of muscular and nervous structures during MIS, which minimizes surgical trauma and facilitates a more comfortable recovery.

These observations are corroborated by Martin's review,^7^ which highlighted that the minimally invasive technique, by reducing soft tissue damage, not only improves functional results, but also significantly reduces postoperative pain and discomfort. These benefits are especially valuable in a context where post-surgical quality of life is a crucial consideration for patients.

### Functional results comparable to open surgery, with shorter hospitalization time

The effectiveness of minimally invasive spinal fusion in terms of functional results is comparable to that of open surgery, with the additional benefit of a shorter hospital stay time. Martin^7^ highlighted that MIS does not compromise long-term functional results, while offering faster recovery, reflected in a shorter hospital stay. This factor is essential to reduce hospital costs and accelerate patients’ return to their daily activities.

In addition, Villavicencio^8^ points out that MIS, by minimizing perioperative complications and accelerating healing, allows patients to return to their normal functional state more quickly, preserving the stability and integrity of the spine. This combination of functional efficacy and practical benefits makes MIS a preferred alternative to open surgery for many patients with degenerative spinal disease.

The bone fusion rates obtained in minimally invasive procedures (MIS) were comparable to those of open surgery (OS), without compromising clinical efficacy. Studies such as those of Martin^7^ and Villavicencio^8^ showed that although the functional results between MIS and OS are similar, the minimally invasive technique presents additional advantages, such as reduced postoperative pain, reduced intraoperative blood loss, shorter hospitalization time and faster recovery.

The results of this meta-analysis indicate that minimally invasive spinal fusion surgery offers several benefits compared to open surgery for degenerative diseases of the spine. The significant reduction in intraoperative blood loss, less postoperative pain, shorter hospitalization time, lower incidence of complications and shorter surgical times make MIS an attractive alternative. In addition, comparable bone fusion rates ensure that clinical efficacy is not compromised. However, it is important to consider the need for specialized training for MIS procedures and the possibility of higher initial costs.

## FINAL CONSIDERATIONS

Based on the analysed data, it is clear that minimally invasive spinal fusion surgery (MIS) presents several advantages compared to open surgery (OS) in the treatment of degenerative diseases of the spine. MIS not only significantly reduces intraoperative blood loss and postoperative pain, but also offers faster recovery, reflected in shorter hospitalization time and lower complication rates. In addition, bone fusion rates comparable to those observed in open techniques ensure that clinical effectiveness is retained without compromising long-term results.

These findings reinforce MIS as a viable and potentially superior alternative to open surgery, especially for patients seeking a less invasive procedure with optimized postoperative results. However, it is essential that healthcare professionals are aware of the learning curve associated with MIS and the specific training needs to ensure the safety and effectiveness of the procedure. As technology and surgical techniques continue to evolve, MIS can further consolidate itself as the preferred approach in the management of degenerative spinal diseases.

## References

[1] Moher D, Liberati A, Tetzlaff J, Altman DG, PRISMA Group (2009). Preferred reporting items for systematic reviews and meta-analyses: the PRISMA statement. PLoS Med.

[2] Higgins JPT, Green S Cochrane Handbook for Systematic Reviews of Interventions Version 5.1.0 2024 [Internet]. The Cochrane Collaboration.

[3] Sterne JAC, Savović J, Page MJ, Elbers RG, Blencowe NS, Boutron I (2019). RoB 2: a revised tool for assessing risk of bias in randomised trials. BMJ.

[4] Liberati A, Altman DG, Tetzlaff J, Mulrow C, Gøtzsche PC, Ioannidis JP (2009). The PRISMA statement for reporting systematic reviews and meta-analyses of studies that evaluate health care interventions: explanation and elaboration. PLoS Med.

[5] Ouzzani M, Hammady H, Fedorowicz Z, Elmagarmid A (2016). Rayyan-a web and mobile app for systematic reviews. Syst Rev.

[6] Zaveri GR, Ford M (2001). Cervical spondylosis: the role of anterior instrumentation after decompression and fusion. J Spinal Disord.

[7] Martin CR, Gruszczynski AT, Braunsfurth HA, Fallatah SM, O'Neil J, Wai EK (2007). The surgical management of degenerative lumbar spondylolisthesis: a systematic review. Spine (Phila Pa 1976).

[8] Villavicencio AT, Burneikiene S, Roeca CM, Nelson EL, Mason A (2010). Minimally invasive versus open transforaminal lumbar interbody fusion. Surg Neurol Int.

[9] Lee N, Shin DA, Yi S, Kim KN, Yoon DH, Ha Y (2018). The efficacy of minimally invasive surgery compared with open surgery for lumbar fusion: a meta-analysis of randomized controlled trials. J Neurosurg Spine.

[10] Goldstein CL, Macwan K, Sundararajan K, Rampersaud YR (2014). Comparative outcomes of minimally invasive surgery for posterior lumbar fusion: a systematic review. Clin Orthop Relat Res.

